# Machine learning models predict lymph node metastasis in patients with stage T1-T2 esophageal squamous cell carcinoma

**DOI:** 10.3389/fonc.2022.986358

**Published:** 2022-09-08

**Authors:** Dong-lin Li, Lin Zhang, Hao-ji Yan, Yin-bin Zheng, Xiao-guang Guo, Sheng-jie Tang, Hai-yang Hu, Hang Yan, Chao Qin, Jun Zhang, Hai-yang Guo, Hai-ning Zhou, Dong Tian

**Affiliations:** ^1^ Department of Thoracic Surgery, Suining Central Hospital, Sunning, China; ^2^ Department of Thoracic Surgery, Affiliated Hospital of North Sichuan Medical College, Nanchong, China; ^3^ Department of Thoracic Surgery, West China Hospital, Sichuan University, Chengdu, China; ^4^ Academician (Expert) Workstation, Affiliated Hospital of North Sichuan Medical College, Nanchong, China; ^5^ Department of Thoracic Surgery, Nanchong Central Hospital, Nanchong, China; ^6^ Department of Pathology, Nanchong Central Hospital, Nanchong, China

**Keywords:** esophageal squamous cell carcinoma, machine learning, lymph node metastasis, predictive model, stage T1-T2

## Abstract

**Background:**

For patients with stage T1-T2 esophageal squamous cell carcinoma (ESCC), accurately predicting lymph node metastasis (LNM) remains challenging. We aimed to investigate the performance of machine learning (ML) models for predicting LNM in patients with stage T1-T2 ESCC.

**Methods:**

Patients with T1-T2 ESCC at three centers between January 2014 and December 2019 were included in this retrospective study and divided into training and external test sets. All patients underwent esophagectomy and were pathologically examined to determine the LNM status. Thirty-six ML models were developed using six modeling algorithms and six feature selection techniques. The optimal model was determined by the bootstrap method. An external test set was used to further assess the model’s generalizability and effectiveness. To evaluate prediction performance, the area under the receiver operating characteristic curve (AUC) was applied.

**Results:**

Of the 1097 included patients, 294 (26.8%) had LNM. The ML models based on clinical features showed good predictive performance for LNM status, with a median bootstrapped AUC of 0.659 (range: 0.592, 0.715). The optimal model using the naive Bayes algorithm with feature selection by determination coefficient had the highest AUC of 0.715 (95% CI: 0.671, 0.763). In the external test set, the optimal ML model achieved an AUC of 0.752 (95% CI: 0.674, 0.829), which was superior to that of T stage (0.624, 95% CI: 0.547, 0.701).

**Conclusions:**

ML models provide good LNM prediction value for stage T1-T2 ESCC patients, and the naive Bayes algorithm with feature selection by determination coefficient performed best.

## Introduction

Esophageal cancer (EC) ranks seventh in annual incidence and sixth in mortality globally, with half of the cases occurring in China, and esophageal squamous cell carcinoma (ESCC) is the predominant histopathological type in Asian populations (1–4). Although esophagectomy with lymphadenectomy remains the gold standard (5–7), endoscopic mucosal resection (EMR) and endoscopic submucosal dissection (ESD) represent new treatment options for early ESCC (5, 8, 9). However, regional lymph node metastasis (LNM) is not uncommon in stage T1-T2 ESCC patients, with a reported occurrence rate ranging from 12.9% to 49.1% (10–14). Controversy still exists about the treatment of early ESCC patients without clinical nodal involvement. For patients with stage T1a ESCC, the decision to perform EMR/ESD is typically influenced by the depth of invasion (DOI), which is associated with the LNM risk (8, 9, 11, 15). Stage T1b-T2 patients with LNM have worse outcomes than those with negative lymph nodes (5–7, 11, 16, 17). Given that LNM negatively impacts survival and prognosis (11, 18, 19), the accurate identification of LNM is highly important to guide a surgeon’s decision about the implementation of endoscopic procedures, surgery, and the subsequent treatment of early-stage ESCC.

Currently, several examination methods are used for preoperative lymph node staging for ESCC. The ability of computed tomography (CT) is unsatisfactory in identifying LNM, with a reported sensitivity, specificity, and accuracy of 39.7%, 77.3%, and 54.5%, respectively (20). Although positron emission tomography-CT (PET-CT) can reliably identify the metastatic lymph nodes that are not enlarged in size, its low sensitivity and high cost remain a concern (21). Endoscopic ultrasonography (EUS) and endobronchial ultrasound (EBUS) show excellent sensitivity but their specificity remains controversial (22, 23). Lymph node biopsy, including EBUS-guided transbronchial needle aspiration (EBUS-TBNA) and EUS-fine-needle aspiration (FNA), may confirm the status of lymph nodes; however, the invasive procedure and post-puncture hematoma limit their wider applications (23, 24). Thus, for preoperatively estimating LNM status, an efficient and precise noninvasive diagnostic approach that is clinically relevant and generalizable is urgently needed.

Machine learning (ML) in artificial intelligence has emerged as a less costly and noninvasive approach to precision medicine in ESCC. In medical research, ML has proven to be an area of interest with many applications, where an acceptable generalization can be attained by using different algorithms and techniques to search an n-dimensional space for a set of medical samples (25). High-dimensional clinical features that are available before and after surgical extirpation of the primary tumor provide a deeper understanding of the LNM that is imperceptible to human eyes. Given the adverse effect of LNM on survival, any decision should be made after careful and accurate preoperative assessment. In this setting, optimizing negative predictive value (NPV), with a focus on minimizing false-negative results, is one of the major objectives of predictive models. ML algorithms can fit fairly complex multinomial interactions or nonlinear relationships, and the resulting predictive accuracy is impressive (25, 26). It has been shown that ML algorithms based on clinical features can identify LNM in other carcinomas (27, 28), without requiring access to and complex preprocessing of imaging data. The current methods for predicting LNM in early ESCC are mainly based on multivariate analysis of clinicopathological characteristics, and lymph node morphology on imaging. However, the literature on clinical feature-based ML prediction models for LNM of T1-2 ESCC is limited. The aim of this study was to develop and externally test ML predictive models for identifying LNM in early-T-stage patients by utilizing clinical features.

## Methods

### Study design and patients

The clinical variables of patients with early-T-stage ESCC were retrospectively collected from three centers (Nanchong Central Hospital, Affiliated Hospital of North Sichuan Medical College, and Suining Central Hospital) between January 2014 and December 2019. The study was registered in the Chinese Clinical Trial Registry (ChiCTR2100051728) and approved by the relevant review boards (Nanchong Central Hospital: 2019-041, Affiliated Hospital of North Sichuan Medical College: 2020ER181-1, Suining Central Hospital: LLSNCH20200027). The informed consent requirement was waived since retrospective, deidentified data were used. Transparent Reporting of a Multivariable Prediction Model for Individual Prognosis or Diagnosis (TRIPOD) was followed in the present study (29).

**Figure 1:**
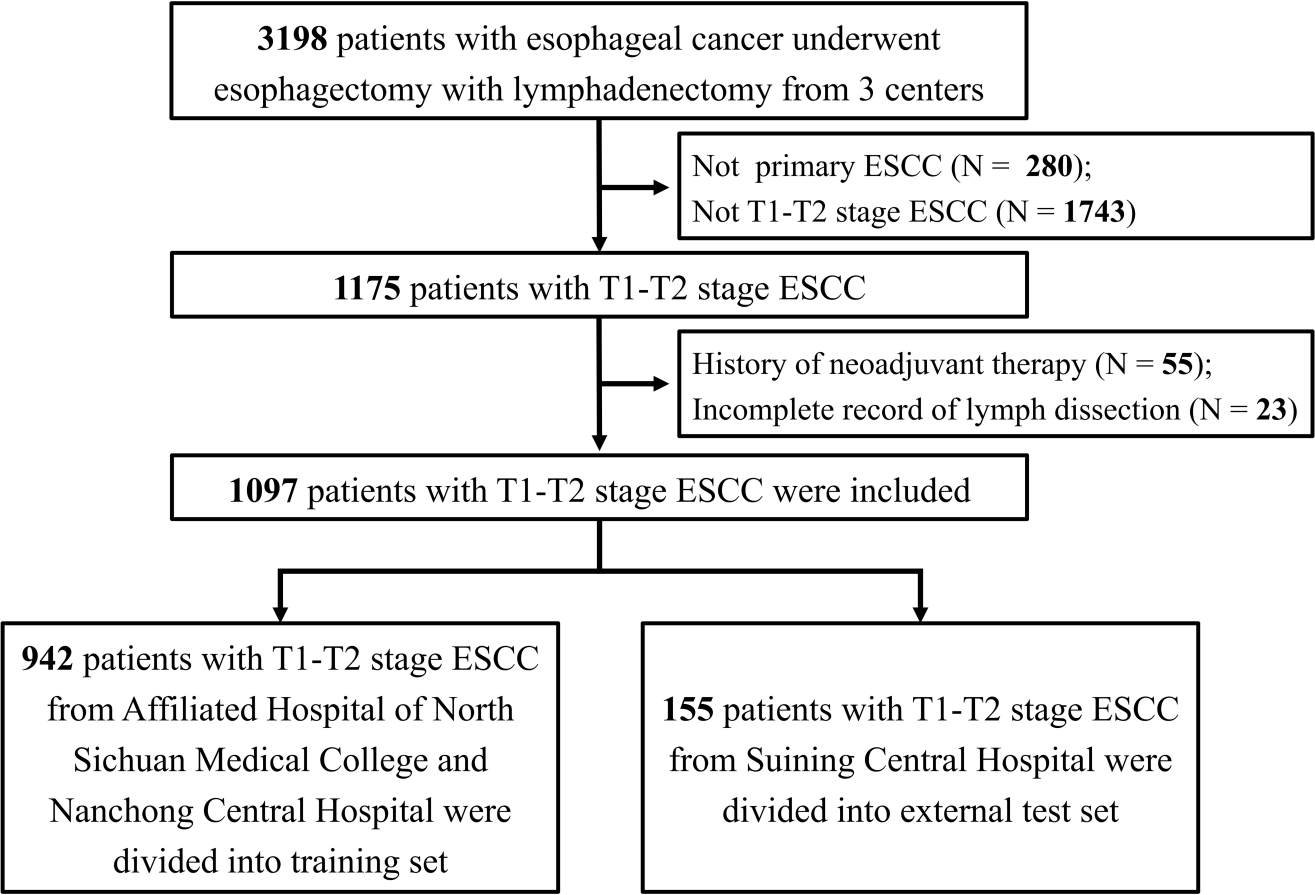
The flow chat for patient inclusion and exclusion. ESCC: esophageal squamous cell carcinoma.

Patients with stage T1-T2 ESCC who had no clinical signs of nodal involvement (cN0) and underwent esophagectomy were identified. The inclusion criteria were defined as follows: (1) patients with primary ESCC; (2) patients aged ≥18 years; (3) McKeown or Ivor Lewis esophagectomy and lymphadenectomy were performed; and (4) pathologically confirmed stage T1 or T2. The following exclusion criteria were applied: (1) multiple primary tumors; (2) neoadjuvant therapy administered prior to surgery; (3) diagnosis of distant metastasis; (4) lymph node examination < 5; and (5) unknown lymph node dissection information. **Figure 1** displays a flow chart of participants included and excluded from the overall study.

### Predictor variables

The characteristics included clinical variables (sex, age, body mass index [BMI], history of surgery, tumor location, and preoperative comorbidities), preoperative hematologic indices (leukocytes, neutrophils, lymphocytes, erythrocytes, hemoglobin, aspartate, alanine, total protein, albumin, globulin, high-density lipoprotein, low-density lipoprotein, lactate, urea, creatinine and glucose), and pathological variables (endoscopic tumor length, tumor size, tumor differentiation and TNM8 T-stage).

### Construction of ML models

Patients from Nanchong Central Hospital and Affiliated Hospital of North Sichuan Medical College were analyzed as the training set. Accounting for potential variation across different research institutes, patients from the Suining Central Hospital were designated as the external test set.

The data analysis involved six feature-selection methods, including random forest (RF), Boruta, least absolute shrinkage and selection operator (LASSO), determination coefficient (DC), relief and recursive feature elimination (RFE). The ML algorithms we evaluated included support vector machine (SVM), generalized boosted regression modeling (GBRM), k-nearest neighbors (KNN), naive Bayes (NB), RF and extreme gradient boosting machine (XGB). These feature selection methods and ML algorithms were common methods, which were introduced in previous studies (27, 30). A total of 36 ML models were developed using the six modeling algorithms and six feature selection techniques for predicting LNM. To validate performance, the bootstrap method was applied with 1,000 repetitions as described in previous literature (31). The area under the receiver operating characteristic (ROC) curve (AUC) was used to assess each model’s overall performance, and a bootstrap resampling methodology was used to assess 95% confidence intervals [CIs]. The sensitivity, specificity, NPV and positive predictive value (PPV) were also evaluated. The best-performing model in the training set was chosen as the final model to predict LNM for the external test set. The AUC of T stage was calculated as a benchmark for the optimal prediction model. Furthermore, to assess the ability of the model to discriminate LNM in patients with different T stages, we conducted a performance evaluation of the optimal model separately for stage T1 and T2 patients in the external test set.

### Statistical analysis

R software version 3.63 was used for the statistical analysis and modeling process. The mean ± standard deviation was used to represent quantitative variables, while the number and percentage were applied to represent categorical variables. The performance of the combined model was evaluated using ROC analysis and AUC calculation.

## Results

### Patient characteristics

In total, 1097 patients were included in our current study. Of these patients, the training set included 942 (85.9%) patients (median age, 65 [41-85] years), and the external test set included 155 (14.1%) patients (median age, 64 [40-80] years). The patient clinical characteristics are summarized in **Table 1**. A total of 294 patients (26.8%) had LNM by final histopathology, including 233 (24.7%) and 61 (39.4%) in the training and external test sets, respectively. The average endoscopic tumor length was 3.8 ± 2.0 cm and 3.7 ± 1.9 cm in the training and external test sets, respectively. The average tumor sizes were 2.8 ± 1.4 cm and 3.3 ± 1.6 cm in the training and external test sets, respectively. In stage T1 and T2 ESCC, the total LN metastasis rates were 16.4% (85/519) and 36.2% (209/578), respectively.

**Table 1:** Clinical characteristics of patients with T1-T2 stage esophageal squamous cell carcinoma.

Characteristics	Training set	External test set
Age (year) (median [range])	65 (41–85)	64 (40-80)
Sex		
Male	665 (69.5%)	117 (75.5%)
Female	287 (30.5%)	38 (24.5%)
BMI	22.9 ± 4.1	21.7 ± 2.5
History of surgery		
Yes	290 (30.8%)	40 (25.8%)
No	652 (69.2%)	115 (74.2%)
Tumor location		
Upper	131 (13.9%)	21 (13.5%)
Middle	606 (64.3%)	78 (50.3%)
Lower	205 (21.8%)	56 (36.2%)
Preoperative complications		
Yes	525 (55.7%)	114 (73.5%)
No	417 (44.3%)	41 (26.5%)
Endoscopic tumor length (cm)	3.8 ± 2.0	3.7 ± 1.9
Tumor size (cm)	2.8 ± 1.4	3.3 ± 1.6
Tumor differentiation		
G1	322 (34.2%)	28 (18.1%)
G2	530 (56.3%)	95 (61.3%)
G3	90 (9.5%)	32 (20.6%)
T stage^†^		
T1a	154 (16.4%)	13 (8.4%)
T1b	294 (31.2%)	58 (37.4%)
T2	494 (52.4%)	84 (54.2%)

BMI, body mass index. ^†^The 8th edition of the UICC and AJCC cancer staging system.

### Predictive performance of machine learning models

**Figure 2:**
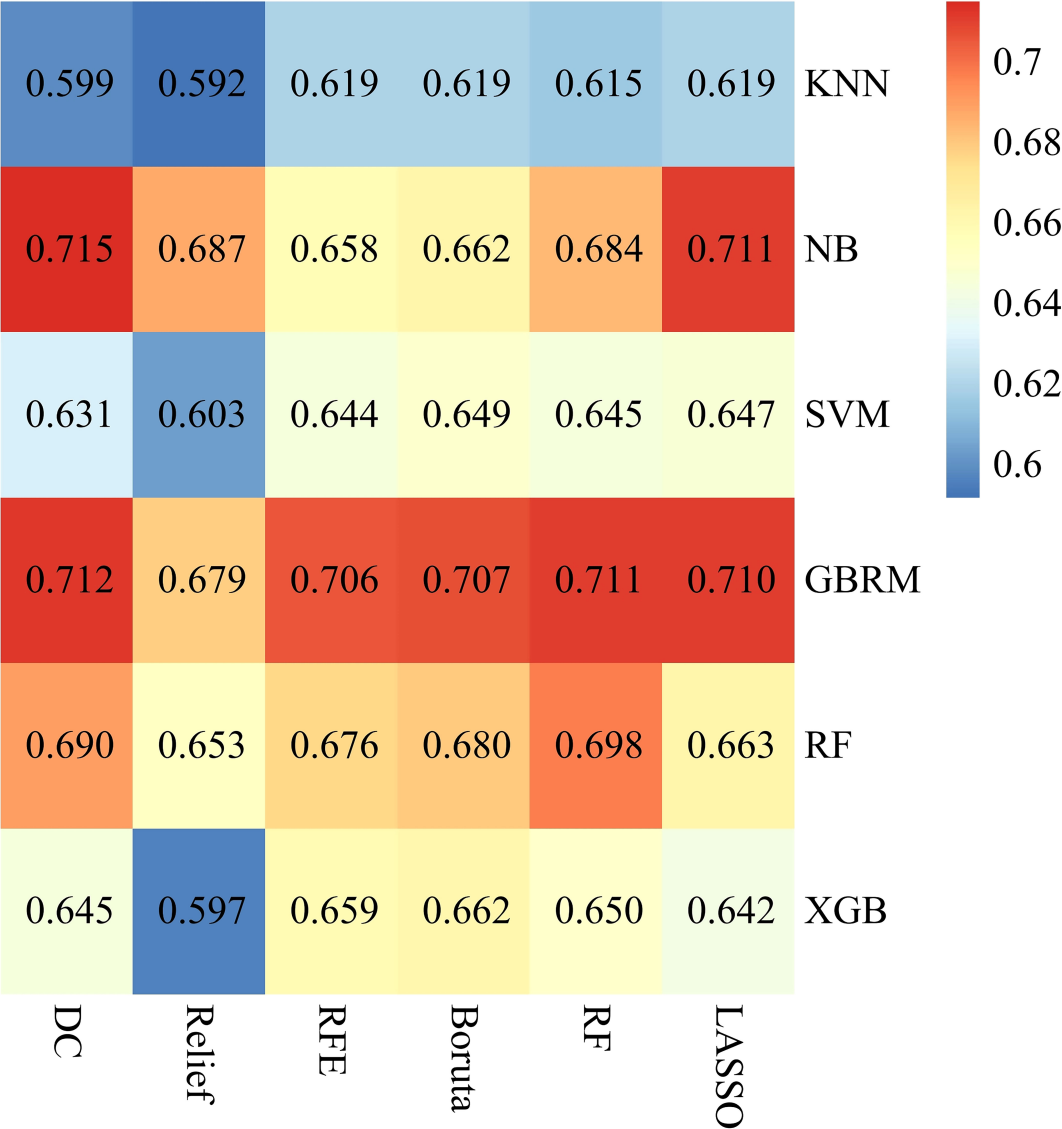
Performance of 36 machine learning models. This Heatmap showed the area under the curve of each machine learning algorithm (columns) with each feature selection method (rows). KNN, k-nearest neighbours; NB, naïve-bayes; SVM, support vector machine; GBRM, generalized boosted regression modeling; RF, random forest; XGB, extreme gradient boosting machine; LASSO, least absolute shrinkage and selection operator; RFE, recursive feature elimination; DC, determination coefficient.

Supervised ML models were trained using patient characteristics to identify patients with LNM. After data preprocessing, 6 discrete features and 20 continuous features in total, listed in **Table 1** and **Supplementary Table 1**, were used for ML modeling. **Figure 2** shows the AUC of each machine learning algorithm (columns) with each feature selection method (rows) in the form of heatmaps. The ML models based on clinical features showed good predictive performance for LNM status, with a median bootstrapped AUC of 0.659 (range: 0.592, 0.715). The NB model using feature selection by determination coefficient exhibited the highest AUC of 0.715 (95% CI: 0.671, 0.763) among all ML models.

### External test for the optimal machine learning model

**Figure 3:**
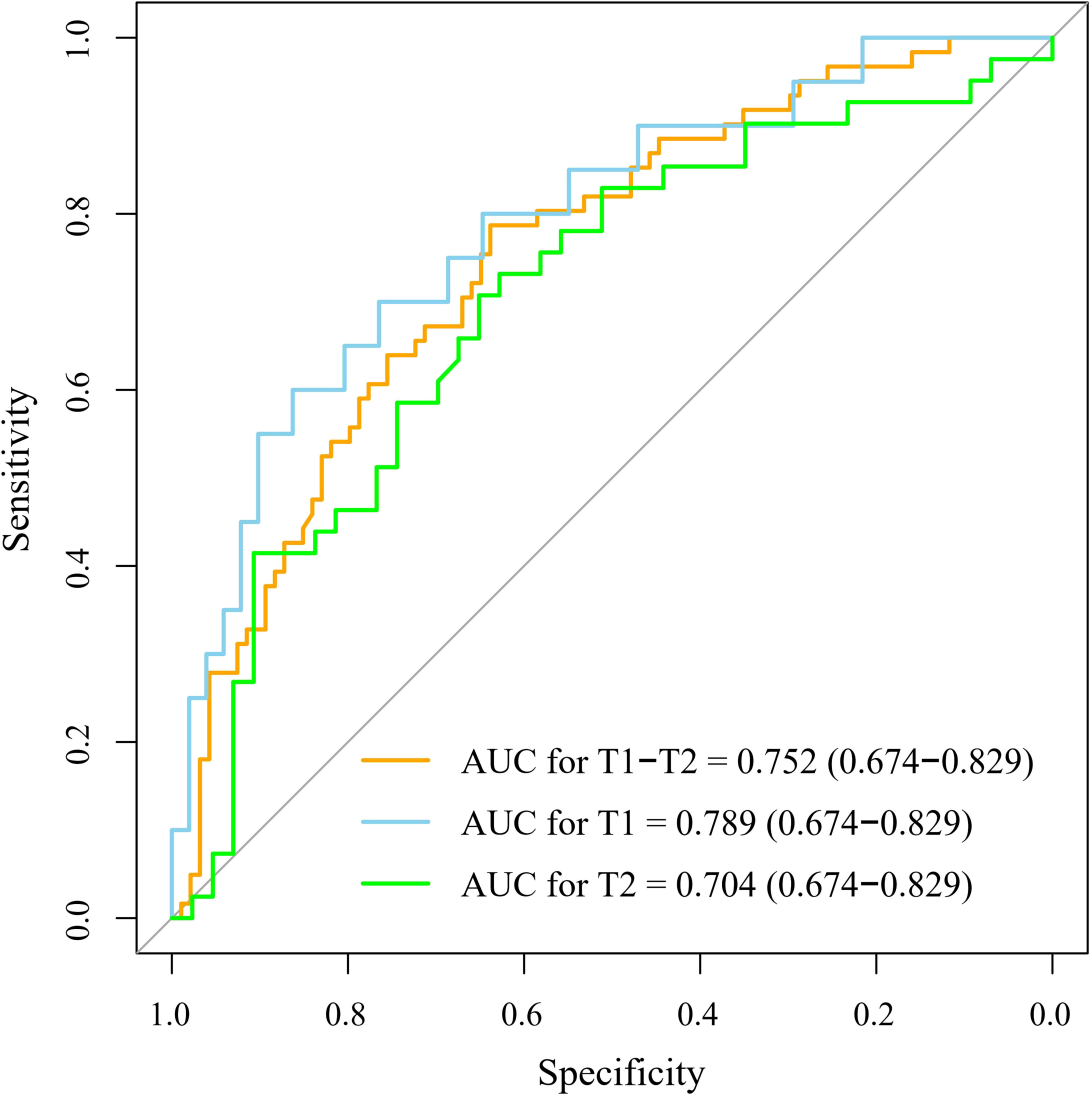
The receiver operator characteristic curve of the optimal machine learning model. The optimal machine learning model exhibited a good performance to predict the LNM for patients with T1-T2, T1, and T2 stage esophageal squamous cell carcinoma. The 95% confidence intervals were showed in the parentheses. AUC, the area under the curve.

**Figure 4:**
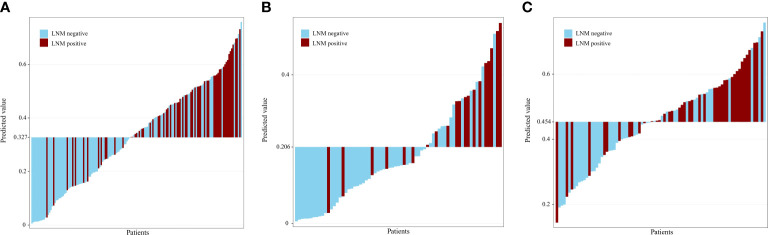
Predicted value for patients with T1-T2 **(A)**, T1 **(B)**, T2 **(C)** stage esophageal squamous cell carcinoma. The predicted value of the naive bayes model could obviously distinguish the different lymph node statuses for patients with T1-T2, T1, and T2 stage esophageal squamous cell carcinoma. LNM, lymph node metastasis.

**Table 2** and **Figure 3** show the results of the optimal model on the external test set. The AUC of the optimal model was 0.787 (95% CI: 0.674, 0.829), which outperformed that of T stage (AUC, 0.624, 95% CI: 0.547, 0.701). The sensitivity, specificity, NPV, and PPV of the optimal model were 78.7%, 63.8%, 82.2% and 58.5%, respectively, which were superior to those of T stage (67.2%, 54.3%, 71.8% and 48.8%, respectively). In both the training and external tests, the performance of the NB model was consistent. We also tested the NB model separately for stages T1 and T2, and the prediction performance was consistent and even better in stage T1. **Figure 4** shows the relative distance of each patient from the decision threshold of the NB model, as determined by their classification probability. The predicted value of the NB model could obviously distinguish the different LNM outcomes of patients with stage T1-T2, stage T1, and stage T2 ESCC.

**Table 2:** Performance of the optimal machine learning model and the T stage.

Model/factor	External validation data	AUC (95% CI)	Sensitivity	Specificity	NPV	PPV
NB model	T1-T2 stage ESCC	0.752 (0.674-0.829)	0.787	0.638	0.822	0.585
T stage	T1-T2 stage ESCC	0.624 (0.547-0.701)	0.672	0.543	0.718	0.488
NB model	T1 stage ESCC	0.789 (0.669-0.901)	0.700	0.765	0.867	0.538
NB model	T2 stage ESCC	0.704 (0.590-0.818)	0.732	0.628	0.711	0.652

NB, Naive Bayes; ESCC, esophageal squamous cell carcinoma; AUC, the area under the curve; CI, confidence interval; NPV, negative predictive value; PPV, positive predictive value.

## Discussion

In this multicenter study, with routinely available clinical data, we developed and validated thirty-six ML models for predicting LNM in stage T1-T2 ESCC patients and revealed 2 main findings. First, the optimal NB model performed well in discriminating LNM with an AUC of 0.715 in the training set and demonstrated similar discrimination on the external test set (AUC 0.753). Second, the predicted value of the NB model could obviously distinguish the different lymph node statuses for patients with stage T1-T2, T1, and T2 ESCC. These novel findings suggest that a clinical feature-based ML model has the potential to be a more effective noninvasive method for identifying LNM in stage T1-T2 ESCC patients.

Lymph node (LN) status is the most important independent prognostic factor in ESCC (11, 32, 33). At present, preoperative assessment of LNM in patients with ESCC is primarily based on CT images using LN size criteria. However, several previous studies showed unsatisfactory discrimination (20, 34). Although EUS, PET-CT, and lymph node biopsy have shown varying degrees of recognition capacity (21–24), high cost and invasive procedures remain a concern. Thus, an efficient and precise noninvasive diagnostic approach that is clinically relevant and generalizable is urgently needed.

The present study showed the feasibility of the ML models for predicting LNM in stage T1-T2 ESCC patients. Traditional logistic regression (LR) analysis showed that tumor length, tumor size, tumor location, T1 substage, differentiation, lymphovascular invasion(LVI), depth of tumor invasion, and macroscopic type were associated with LNM occurrence (11, 14, 35–39). Multivariate analysis demonstrated that poor differentiation, LVI, depth of tumor invasion, T1 substage, high-density lipoprotein cholesterol (HDL-C) level, and preoperative alanine aminotransferase/aspartate aminotransferase ratio (LSR) were significant independent risk factors for LNM (11, 14, 35, 37–39). Hence, the twenty-six routinely available features extracted from patients, including clinical variables, hematological indicators and pathological variables, are feasible for predicting LNM. In most cases, prediction models have been developed based on input features considered significant by clinicians (14, 35, 40). Through human assumptions, this approach may limit the choice of input features and result in biasing. Selecting a large set of input features and using ML models to select those that perform the best may mitigate the issue to some extent (30). Some of these may not correlate with those deemed to be most important by clinical professionals and may highlight features that were previously not considered. Our models may prove useful in implementing personalized treatment stratification and close surveillance in the future by using extensive clinical features.

The literature on ML models for LNM prediction in early ESCC constructed using readily available clinical data is limited. An artificial neural network (ANN) based on clinical features was built for superficial esophageal squamous cell carcinoma (SESCC), and its ability to predict LNM was compared with that of a traditional LR model (41). The ANN model outperformed the LR model with respect to the AUC, specificity, PPV, and accuracy (41). It may be a valuable tool, especially for determining the need for additional treatment after ESD procedures. However, only one ML algorithm and feature selection method were used in their work. In our study, we compared multiple feature selection methods and ML algorithms and determined the optimal model using the bootstrap method. NB is a simple but powerful classification method widely used within ML technique. This probabilistic classifier has been proven to be highly professional and based on solid mathematical principles, with the advantages of fast predictions, adaptability to different numbers of datasets, and quickly updates as new training data becomes available (42). In addition, a concern demonstrated in their work is the lack of external testing regarding the predictive performance of their models. In this study, we confirmed the performance of our optimal NB model in an independent external test dataset. The results were consistent in both the training and external test sets, suggesting that the model was not overfitted. Building on this work, using multiple ML algorithms with feature selection methods for our specific dataset is a robust approach to select the most suitable model.

T stage is an independent risk factor for LNM of ESCC (43). The rate of LNM differs substantially between different T stages. In this study, the LNM rates of stage T1 and T2 were 16.4% and 36.2%, respectively, which was consistent with the reported incidence (10, 35, 37, 44). To distinguish the ability of the model to discriminate LNM in patients with T1 and T2 stages, we tested the optimal model on different T stages of the external test set. The optimal NB model exhibited good performance in predicting LNM for patients with T1-T2, T1, and T2 stage ESCC. In addition, we constructed grayscale histograms to distinguish benign and malignant LNs in different T stages. The predicted value of the NB model could obviously distinguish the different lymph node statuses for patients with T1-T2, T1, and T2 stage ESCC. The current study showed that a stable classification model with a similar AUC value in stages T1-T2, T1, and T2 is useful to differentiate metastatic from nonmetastatic lymph nodes.

Other strengths of this study include its large sample size and multicenter design. Models were developed utilizing readily available clinical data without complicated preprocessing of imaging data. Various features were examined, including patient demographics, physical fitness, and tumor characteristics. In low-resource settings, this approach to modeling using local clinical datasets could be replicated across health systems to benefit the treatment stratification and subsequent surveillance of patients with early-stage ESCC at risk of LNM. Furthermore, to demonstrate that clinical feature-based models have real benefits, they should be compared to more advanced models based on clinical features. Our methodology could provide the foundation for such models.

The limitations of the present study include three aspects. First, it is a retrospective design article. A prospective external test of the NB model in a large cohort population would be necessary for generalizing the findings. Second, the amount of dataset information is inadequate. Several features were omitted. Commonly, a larger amount of data will improve the confidenceand performance of our model. Third, considering the clinical features alone in this study, the number of the models established was insufficient, and the AUCs were not high enough. An integrated radiomics analysis of the primary tumor and lymph nodes could potentially improve prediction performance.

## Conclusions

In this study, we developed prediction models for identifying LNM in stage T1-T2 ESCC by comparing multiple ML algorithms and feature selection methods. These models achieved reasonable prediction performance. The optimal NB model demonstrated similar discrimination in the training and external test sets. Although advanced models may surpass this approach, the use of routinely available clinical data can be beneficial. Validated and externally tested, this robust and ready-to-use ML model sets the stage for future clinical trials involving the risk stratification of LNM for early ESCC.

## Data availability statement

The original contributions presented in the study are included in the article/**Supplementary Material**. Further inquiries can be directed to the corresponding authors.

## Ethics statement

The studies involving human participants were reviewed and approved by The Ethics Committee of Nanchong Central Hospital, Affiliated Hospital of North Sichuan Medical College, and Suining Central Hospital. Written informed consent for participation was not required for this study in accordance with the national legislation and the institutional requirements.

## Author contributions

DL and LZ: methodology, data collection, data analysis, and original draft. H-JY: methodology, statistical analysis, data visualization, and manuscript editing. YZ, XG, ST, HH, and HY: data collection and manuscript editing. CQ, JZ, and HG: data collection and manuscript editing. HZ and DT: conceptualization, project administration, and manuscript editing. All authors had access to the data and reviewed the manuscript.

## Conflict of interest

The authors declare that the research was conducted in the absence of any commercial or financial relationships that could be construed as a potential conflict of interest.

## Publisher’s note

All claims expressed in this article are solely those of the authors and do not necessarily represent those of their affiliated organizations, or those of the publisher, the editors and the reviewers. Any product that may be evaluated in this article, or claim that may be made by its manufacturer, is not guaranteed or endorsed by the publisher.
