# Supplementary material for: Machine learning models predict lymph node metastasis in patients with stage T1-T2 esophageal squamous cell carcinoma
Source: Front Oncol. 2022 Sep 8;12:986358. doi: 10.3389/fonc.2022.986358 (PMC9496653; doi:10.3389/fonc.2022.986358)
Supplement: Supplementary file 1 [file Table_1.docx]

**Supplementary Table 1. Hematological characteristics of patients with T1-T2 stage esophageal squamous cell carcinoma.**

| **Characteristics** | **Training set** | **External test set** |
| --- | --- | --- |
| Leukocytes (10^9^/L) | 6.4 ± 3.0 | 6.2 ± 2.4 |
| Neutrophils (10^9^/L) | 4.1 ± 2.5 | 3.9 ± 2.3 |
| Lymphocytes (10^9^/L) | 1.8 ± 1.3 | 1.5 ± 0.6 |
| Erythrocyte (10^12^/L) | 4.2 ± 0.5 | 4.3 ± 0.6 |
| Hemoglobin (g/L) | 128.6 ± 15.8 | 132.3 ± 14.7 |
| Aspartates (U/L) | 25.0 ± 21.4 | 24.7 ± 9.7 |
| Alanine (U/L) | 19.3 ± 21.2 | 21.9 ± 14.9 |
| Total protein (g/L) | 68.7 ± 6.7 | 68.9 ± 6.3 |
| Albumin (g/L) | 40.3 ± 5.2 | 40.1 ± 4.3 |
| Globulin (g/L) | 28.8 ± 4.8 | 28.9 ± 4.4 |
| High-density-lipoprotein (mmol/L) | 1.3 ± 0.4 | 1.4 ± 0.3 |
| Low-density-lipoprotein (mmol/L) | 2.7 ± 0.8 | 2.3 ± 0.6 |
| Lactate (mmol/L) | 2.6 ± 0.8 | 2.3 ± 0.7 |
| Urea (mmol/L) | 5.7 ± 2.7 | 5.3 ± 1.5 |
| Creatinine (umol/L) | 64.6 ± 17.2 | 57.1 ± 16.4 |
| Glucose (mmol/L) | 5.2 ± 1.9 | 5.0 ± 2.2 |

All continuous variables were displayed by mean ± standard deviation.
